# Downregulation of Parahippocampal Copper Chaperone for Superoxide Dismutase in Alzheimer’s Disease

**DOI:** 10.3390/brainsci15030216

**Published:** 2025-02-20

**Authors:** Nicholas Sanchez, Danilo S. Boskovic, Charles W. Diamond, Timothy W. Lyons, Salvador Soriano, Wolff M. Kirsch

**Affiliations:** 1 Division of Biochemistry, Department of Basic Sciences, School of Medicine, Loma Linda University, Loma Linda, CA 92350, USA; nsanchez@students.llu.edu (N.S.); dboskovic@llu.edu (D.S.B.); wkirsch@llu.edu (W.M.K.); 2 Neurosurgery Center for Research, Training and Education, School of Medicine, Loma Linda University, Loma Linda, CA 92350, USA; 3 Department of Earth and Biological Sciences, School of Medicine, Loma Linda University, Loma Linda, CA 92350, USA; 4 Department of Earth and Planetary Sciences, University of California, Riverside, CA 92521, USA; charles.diamond@ucr.edu (C.W.D.); timothy.lyons@ucr.edu (T.W.L.); 5 Laboratory of Neurodegenerative Diseases, Department of Pathology and Human Anatomy, School of Medicine, Loma Linda University, Loma Linda, CA 92350, USA

**Keywords:** copper, Alzheimer’s disease, CCS, ATOX1, DCTN4, dynactin, p62, reactive oxidative stress, copper transport, neurotoxicity

## Abstract

Background/Objectives: Proper regulation of copper is essential for maintaining neuronal stability and is facilitated by several chaperone proteins, protecting cells from oxidative damage that would otherwise be caused by improperly regulated copper ions. Oxidative stress, resulting from such dysregulation, is hypothesized to play a significant role in the pathogenesis of Alzheimer’s disease (AD). Methods: In this study, we evaluated the concentrations of the copper chaperones CCS, DCTN4, and ATOX1 in control and AD cases via Western blotting and ELISA, and quantified the copper concentrations in fractionated neurons using ICP-MS. Results: Our findings reveal a significant reduction in CCS levels in AD cases (*p* = 0.0085), with a progressive decline observed with advancing age. This decline was more pronounced in women, although the difference did not reach statistical significance (*p* = 0.0768). No significant differences were observed in copper concentrations within synaptosomal (*p* = 0.3869) or cytosolic fractions (*p* = 0.4461) between the AD and control cases. Additionally, comprehensive analyses of the effects of sex and age showed no significant impact on the levels of copper chaperones or copper distribution across cellular compartments. Conclusions: These results suggest a strong association between reduced CCS levels and AD pathology, highlighting a potential role for CCS in the redistribution of copper ions within neurons. This redistribution may contribute to oxidative stress and neuronal dysfunction, offering new insights into the mechanisms underlying AD pathogenesis.

## 1. Introduction

Alzheimer’s disease (AD) is a complex neurological disorder with multiple competing hypotheses regarding its etiology and pathophysiology. This complexity has resulted in inconsistent findings across studies. One area of growing interest is the potential role of oxidative stress in AD pathogenesis, particularly focusing on proteins that regulate reactive oxygen species (ROS). Within this framework, the regulation of copper oxidation has emerged as a significant area of investigation. Copper (Cu) is an essential trace element needed for brain function [1]. However, its precise role in AD remains uncertain. Several studies have reported elevated levels of both total and free copper in the serum of AD patients compared to healthy controls, suggesting a potential link between copper homeostasis and AD pathology, although the exact nature of this relationship requires further elucidation [2,3,4].

Copper exists in two valence states: In its reduced form, Cu(I) is the primary state used for intracellular transport and is tightly regulated by cellular buffering systems. Conversely, Cu(II), the oxidized form, serves as a vital cofactor for many enzymes, enabling a wide array of biochemical reactions. It cycles between these two forms during redox reactions, requiring strict regulation for the maintenance of proper cellular function [1,5,6]. When not involved in catalytic functions, copper can participate in Fenton-type reactions, cycling between Cu(II) and Cu(I) oxidation states. This process generates ROS, including the highly reactive hydroxy radical (HO•), and it is linked to both normal aging and neurodegenerative disorders, including AD [7,8,9,10]. In normal aging, oxidative damage accumulates gradually, leading to a slow decline in cellular function and repair. However, in AD, this process is accelerated [11], and it has been reported that AD patients show reduced levels of copper-dependent enzymes [12,13,14], negatively affecting energy production, as copper is essential for cytochrome c oxidase, as well as antioxidant mechanisms, as many copper-dependent enzymes play key roles in responses to oxidative stress [15].

**Figure 1:**
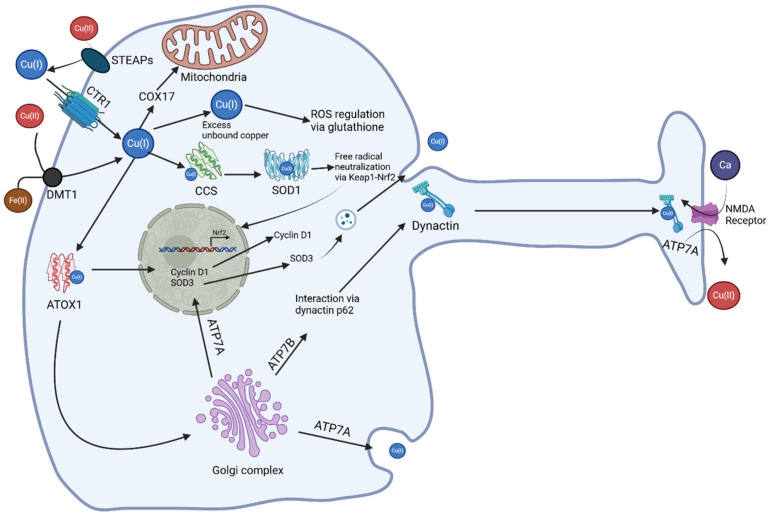
Copper transport within a generalized neuron. Copper needs to be reduced by the membrane-bound protein STEAP and enters the cell via the membrane transporter CTR1, DMT1 with Fe(II). When inside, copper chaperones will take the loose copper and shuttle it where it is needed. COX17 takes copper to the mitochondria, where it binds to SCO1 and COX11. CCS takes copper to SOD1 in the cytosol. ATOX1 takes copper to the Golgi complex. Cu-ATPases use ATP hydrolysis to then bind Cu and take it out of the cell via vesicles to be delivered to ceruloplasmin in human serum, or to the nucleus where it activates protein folding for Cyclin D1 or SOD3. Excess loose copper is bound by glutathione. ATP7B delivers copper to the axonal transporter dynactin to be taken to the axon, where the ion exits the cell in exchange for calcium ions. ATOX1, antioxidant protein 1; ATP7A, ATPase copper transporting alpha; ATP7B, ATPase copper transporting beta; CCS, copper chaperone for Superoxide Dismutase; COX17, Cytochrome c oxidase copper chaperone 17; CTR1, copper transporter 1; DMT1, divalent metal transporter 1; NMDA, N-methyl-D-aspartate; SOD1, superoxide dismutase 1; SOD3, superoxide dismutase 3; STEAP, six transmembrane epithelial antigen of prostate.

Figure 1 depicts an overall view of copper homeostasis in neurons. Specifically, copper enters neurons through the cell membrane via the CTR1 transporter, which facilitates the majority of cellular copper uptake [16,17]. Once inside the cytoplasm, copper follows various pathways depending on cellular needs. It can bind to thiol-containing metabolites such as glutathione or be directed by specific copper chaperones. For example, the Antioxidant protein 1 (ATOX1) copper chaperone shuttles copper to the Golgi complex where ATPases bind to it during ATP hydrolysis [17]. These ATPases can either expel copper from the cell via exocytosis or take it to the nucleus where it activates protein folding [17]. Additionally, ATOX1 may independently deliver copper to the nucleus. Unbound copper can also interact with the copper chaperone for Superoxide Dismutase 1 (CCS), which exclusively delivers copper to Superoxide Dismutase 1 (SOD1) [18]. This process activates and supports the neutralization of free radicals via Keap1-Nrf2 signaling [19,20,21,22,23]. Copper can be transported along the axon and released into the synaptic cleft with the assistance of the dynactin subunit p62, which facilitates its movement through retrograde transport mechanisms [24,25,26]. Upon reaching the synapse, copper is released into the synaptic cleft via ATP7A, a copper-transporting ATPase that is activated by calcium influx through NMDA receptors [27,28].

The presence of copper must be tightly regulated within the neuron by copper chaperones to prevent neuronal damage, since dysregulated copper has been strongly implicated in various forms of dementia due to its role in the rapid generation of harmful oxidative stress [29,30,31]. For instance, CCS deficiency has been associated with amyotrophic lateral sclerosis (ALS) [32,33,34]. Similarly, ALS has also been associated with the loss of dynactin machinery, potentially leading to downstream effects such as mitochondrial dysfunction and the aggregation of mutant SOD1 [35]. Another copper carrier, ATOX1 plays a role in protecting neuronal viability under oxidative stress by regulating copper transport to ATPases such as ATP7A and ATP7B [36]. A deficiency in ATOX1 disrupts this copper modulation, contributing to conditions like Menkes disease (caused by defective ATP7A) and Wilson’s disease (characterized by secondary copper overload due to loss of ATP7B function) [37]. A common feature of these copper chaperones is that their dysregulation tends to exacerbate oxidative stress. Such imbalances can lead to visible copper deposits in brain regions such as the hippocampus, temporal gyrus, or substantia nigra [6].

In this study, we measured the expression levels of the copper chaperones CCS, DCTN4, and ATOX1 in control and AD cases, and quantified copper concentrations in different neuronal fractions to help identify potential pathways leading to oxidative stress and neuronal death in AD. Using a combination of established techniques and a novel approach, we report a significant reduction in CCS levels in AD cases, suggesting a strong association between CCS dysfunction and AD pathology.

## 2. Materials and Methods

### 2.1. Brain Tissue

Tissue specimens were acquired from the NIH Brain & Tissue Repository-California, Human Brain & Spinal Resource Center, VA West Los Angeles Medical Center, Los Angeles, California, which is supported in part by the National Institutes of Health and the US Department of Veterans Affairs. This brain bank operates under the IRB Protocol Number PCC#: 2015-060672, VA Project #: 0002, and is managed by the Department of Veterans Affairs—Los Angeles. Brains are removed from the calvarium, packed in wet ice, and transported to laboratories within 24 h of death. Sections are flash-frozen and stored at −80 °C. Patients range from 65 to 95-years old. All samples were from Caucasian patients for demographic consistency. The control group consisted of thirteen patients who received no diagnosis of AD. Fifteen patients were in the AD group, with AD characterized as Braak and Braak Stage VI (Table 1). All sections were taken from the parahippocampal region in Brodmann’s Area 21, an area which sees a severe loss of neurons in the later stages of AD [38]. Samples ranged in mass from 0.6 to 1.3 g and were obtained with a postmortem delay ranging between 4 and 28 h (average 13). Neuropathic changes were described as pure AD changes without co-morbidities related to cerebral amyloid angiopathy. Further information on the cases used is available in Supplemental Table S1.

### 2.2. Protein Extraction

For the analysis of dynactin p62 and CCS, human brain samples were homogenized at 10% (*w*/*v*) in a lysis buffer containing 50 mM Tris-HCl, 5 mM EGTA, 10 mM EDTA, and Pierce Protease and Phosphatase Inhibitor Mini Tablets. The homogenization was performed on ice using a Dounce homogenizer, with 10 strokes using a loose pestle followed by 10 strokes with a tight pestle. The homogenate was then centrifuged at 2000× *g* for 2 min, and the supernatant was collected into a new tube. Aliquots were either used immediately or stored at −80 °C for later use. To measure ATOX1, human brain samples were homogenized in a buffer at 1.5X (*w*/*v*) containing 10 mM Tris-HCl, SDS, leupeptin, and protease inhibitors [39]. The homogenization was performed on ice using a Dounce homogenizer, with 10 strokes using a loose pestle followed by 10 strokes with a tight pestle. The homogenate was then subjected to sonication using a Branson Sonifier^®^ Cell Disruptor Model SLPe for 6 pulses at 20% amplitude, with 5 s on and 10 s off per pulse. After sonication, the homogenate was centrifuged at 15,700× *g* for 10 min at 4 °C to clarify the solution. The supernatant was collected and either used immediately or stored at −80 °C for future analysis.

### 2.3. Western Blot

Protein quantification of dynactin p62 and CCS was analyzed via Western blot. Samples were heated to 70 °C for 10 min. Proteins were run on a Novex™ Tris-Glycine Mini Protein Gels, 10–20%, 1.0 mm, in WedgeWell™ format (Thermo Fisher XP10202BOX, Waltham, MA, USA). Gels were run at 120 V for 120 min. Protein bands were transferred to Supported Nitrocellulose Transfer Membrane 0.45 μm (GVS 1212602) at 120 V for 45 min. The protein transfers were verified on membrane via 0.1% Ponceau S staining. Blocking was achieved using 5% milk for 1 h. Incubation with primary antibodies (Abcam Waltham, MA, USA and Santa Cruz Biotech, Dallas, TX, USA) was carried out overnight at −20 °C. β-actin was used as the loading control. Secondary antibodies (1:1000 dilution) were incubated for 1 h at room temperature, followed by incubation in TBS-T three times for 5 min each. Membranes were imaged using the Li-Cor Odyssey CLx-2506 Imaging System (Lincoln, NE, USA), using Image Studio Ver 5.5 for acquisition. Appropriate wavelengths for each antibody (IRDye 680RD and IRDye 800CW) were used for scanning. Gel quantification and analysis were carried out using ImageJ 1.54i and Microsoft Office Excel (Version 2405) utilizing the methods outlined by Stael et al. [40].

### 2.4. ELISA

Protein quantification for ATOX1 was analyzed via competitive ELISA test kits purchased from MyBioSource.com (Catalog MBS7217810). Results were read spectrophotometrically via a microplate reader (Bio-Rad 1681130 iMark™ Microplate Absorbance Reader, Hercules, CA, USA) using Microplate Manager Software 6. Absorbance was read at 450 nm. Results were calculated via 4-parameter logistic curve using AAT Bioquest software [41], accessed in February 2024. Microsoft Excel was used for data analysis utilizing the curve results from these online calculations.

### 2.5. Synaptosomal Isolation

Synaptosome isolation was accomplished using Syn-PER Synaptic Protein Extraction Reagent (Thermo Fisher 87793). Brain samples were homogenized in ice using a Dounce homogenizer 10 times in 10% homogenization buffer with protease inhibitors (Pierce Protease and Phosphatases Inhibitor Mini Tablets, EGTA, Trypsin, Pepstatin). The sample was first centrifuged at 1200× *g* for 10 min at 4 °C, followed by a second centrifugation at 15,000× *g* for 20 min at 4 °C to isolate the synaptosomal fraction, cytosolic fraction, and remaining homogenate. Synaptosome isolation was confirmed via Western blotting using synaptic marker postsynaptic density protein 95 (PSD95), cytosolic marker cyclin-dependent kinase 5 (CDK5), and nuclear marker histone deacetylase 2 (HDAC2).

### 2.6. Copper Quantification via Inductively Coupled Plasma Mass Spectrometry (ICP-MS)

To analyze total copper, each fraction isolated from the tissue samples was digested in 70% nitric acid overnight. Following nitric treatment, the samples were heated to 80 °C for 20 min, allowed to cool, diluted in 10% hydrogen peroxide, heated to 70 °C for 15 min, and allowed to cool again until no insoluble fraction remained. Samples were analyzed via ICP-MS (Agilent 7500ce, Santa Clara, CA, USA). The standard curve ranged from 0 to 10 ppb using NIST 1640a as the reference standard. Hydrogen (H_2_) readings were deemed more reliable due to the consistent adherence to the standard curve.

### 2.7. Statistical Analysis

All experimental data are presented as the averages of three independent experiments. Statistical analysis was carried out using GraphPad Prism 10.3.1 (509). A two-tailed *t*-test with Welch’s correction was used to assess statistical significance between the two groups, while two-way ANOVA with Tukey’s multiple comparisons test was applied for analyses involving multiple variables. Statistical significance was set at *p* < 0.05 for *, and *p* < 0.01 for **.

## 3. Results

**Figure 2:**
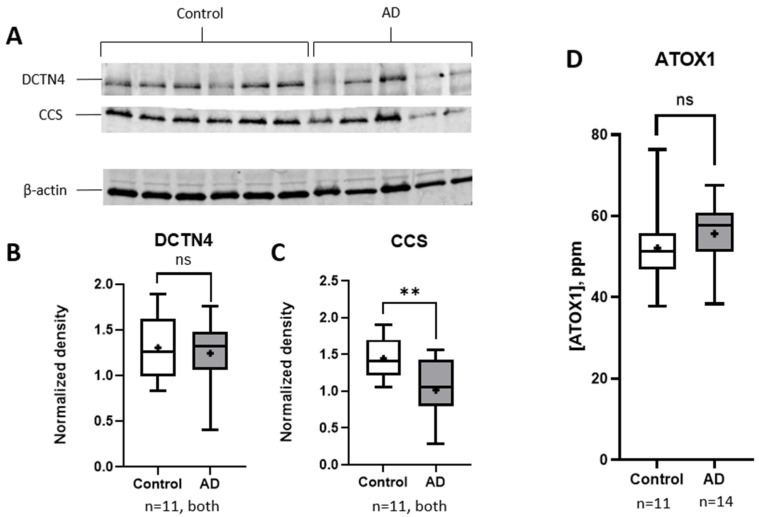
Copper chaperone presence in human brain samples. Panel A shows a representative blot; quantified data for all samples are shown in Panels B–C. (**A**) The Western blot shows lower expression of CCS in cases diagnosed with Alzheimer’s disease. An expanded view of the entire membrane in both the 680 nm and 800 nm channels is available in Supplemental Figure S1. (**B**–**D**) Overall results for the copper chaperones are represented via box and whisker plots, with lines representing the median value and the mean represented by +. (**B**,**C**) Comparison between sample populations shows no significant changes in DCTN4 expression between control cases and Alzheimer’s cases (*p* = 0.6797). CCS was significantly reduced in brains affected by Alzheimer’s disease (*p* = 0.0085). (**D**) ATOX1 in homogenized brains measured via competitive ELISA showed no statistical significance between both groups (*p* = 0.3318). The N values for each group are shown in the figure. Statistical significance was set at *p* < 0.01 for **, and ns = not significant. A representation of copper chaperone data as scatter dot plots is provided in Supplemental Figure S2. AD, Alzheimer’s disease; ATOX1, antioxidant protein 1; CCS, copper chaperone for Superoxide Dismutase; DCTN4, dynactin subunit 4.

To compare the expression levels of the copper chaperones DCTN4 and CCS in the control and AD samples, whole-brain homogenates were analyzed by Western blotting. Figure 2A displays a representative blot, while Figure 2B,C provides a full quantitative analysis of all samples.

Each experiment used β-actin as a loading control. In some cases, the membranes were cut into strips to prevent potential cross-reactivity between antibodies. An analysis of the normalized densities of the Western blots, as described in the Materials and Methods section, revealed a significant reduction in the copper chaperone CCS in cases diagnosed with AD compared to the controls (95% CI: −0.7395 to −0.1239, *p* = 0.0085) (Figure 2C). The CCS levels were found to be 25% lower in the brains of AD patients (1.060 normalized density units) compared to healthy controls (1.405 units), which was consistent with the expectations of copper ion dysregulation. In contrast, the expression of the copper chaperone DCTN4 showed no significant difference between the AD and control cases, with normalized densities of 1.243 and 1.303, respectively (Figure 2B). A two-tailed *t*-test with Welch’s correction confirmed the lack of significance (95% CI: −0.3575 to 0.2375; *p* = 0.6797).

To quantify the ATOX1 expression levels in the control and AD samples, we employed ELISA, as the available antibodies did not produce consistent results in the Western blot analysis. As illustrated in Figure 2D, no statistically significant differences in ATOX1 expression levels were observed between the AD and control groups, with normalized densities of 55.68 and 52.08, respectively. (95% CI: −3.958 to 11.15; *p* = 0.3318).

**Figure 3:**
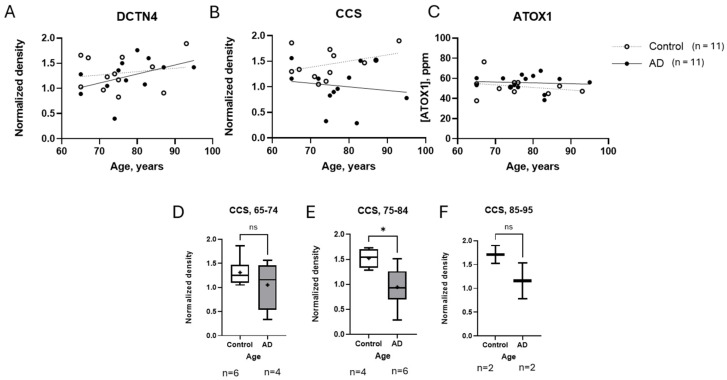
Age-associated changes in copper chaperone levels in AD and control cohorts. (**A**–**C**) Levels of copper chaperones DCTN4, CCS, and ATOX1 in control and AD groups from ages 65 to 97. CCS exhibits an age-dependent decline in AD patients compared to controls (*p* < 0.05, two-way ANOVA with Tukey’s post hoc test). (**D**–**F**) Age-stratified analysis of CCS levels. Cohorts were divided into three age groups: 65–74 (n = 6 controls, 4 AD), 75–84 (n = 4 controls, 6 AD), and 85–95 (n = 2 controls, 2 AD). Significant CCS reductions in AD patients were observed in the 75–84 age group (*p* = 0.0178). Non-significant trends in the 85–95 cohort likely reflect the limited sample size (n = 2 per group). Boxplots show interquartile ranges (boxes), median (central line), and mean (+ symbol). Whiskers extend to minimum/maximum values. Statistical significance was set at *p* < 0.05 for *, with ns = not significant. Full age-stratified data for all chaperones are in Supplemental Figure S3. AD, Alzheimer’s disease; ATOX1, antioxidant protein 1; CCS, copper chaperone for Superoxide Dismutase; DCTN4, dynactin subunit 4.

Given the significant decrease in CCS observed in the AD cases, we expanded our investigation to examine the influence of other demographic factors on the copper chaperones under study. We focused particularly on age, as it is the most important risk factor for AD. Aging is associated with numerous measured metabolic changes, which could potentially impact copper homeostasis and chaperone function [42]. Figure 3A–C present the expression levels of DCTN4, CCS, and ATOX1 across a range of ages. DCTN4 trended upward with age with no obvious differences between the healthy and AD groups (Figure 3A). ATOX1 showed a slight decrease with age, with no distinct difference between the control and AD groups (Figure 3C). Interestingly, CCS trended downwards in the brains of patients with AD with advancing age, while in healthy individuals a slight increase was observed (Figure 3B). To assess whether age significantly influenced the CCS measurements, we divided the cases into three age groups (65–74, 75–84, and 85–95 years), and performed Welch’s *t*-tests for each group (Figure 3D–F. The number of samples in each group was n(controls, AD) = (6,4), (4,6) and (2,2), respectively. In all three age groups, the CCS medians and means were lower in the AD samples. However, in the 75–84 age group, there was a significant difference in the CCS levels (*p* = 0.0178), revealing a distinct trend in opposing directions between the control and AD cases (Figure 3E). Although a similar trend was also observed in the 85–95 age group, the low sample size (n = 2 for both control and AD cases) limited the statistical power of this result, and the *p*-value did not reach significance (*p* = 0.3544) (Figure 3F).

**Figure 4:**
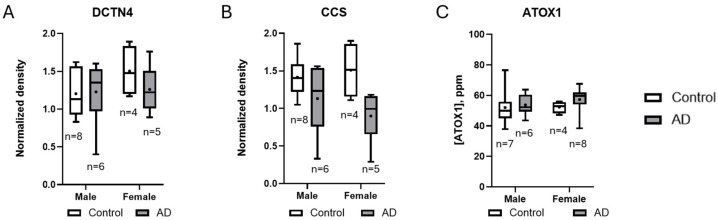
Sex-stratified analysis of copper chaperone levels in control and Alzheimer’s disease (AD) cohorts. Lines represent median values, with + symbols indicating means. (**A**) DCTN4 showed no significant changes. (**B**) A non-significant trend toward reduced CCS levels was observed in female AD patients compared to female controls (*p* = 0.0768), while male cohorts showed no such trend (*p* = 0.4897). (**C**) No changes in ATOX1 measurements. N values for each group are shown. There was no sex-dependent effect on copper chaperone presence between male and female cases. Representation of data as scatter dot plots is provided in Supplemental Figure S4. AD, Alzheimer’s disease; ATOX1, antioxidant protein 1; CCS, copper chaperone for Superoxide Dismutase; DCTN4, dynactin subunit 4.

Using two-way ANOVA, we also evaluated the effect of sex on copper chaperone levels, with between-group differences analyzed using Tukey’s multiple comparisons test (Figure 4). Overall, sex did not have a statistically significant impact on the levels of the three copper chaperones measured. The most notable difference was observed in CCS measurements among females, though this did not reach statistical significance (*p* = 0.0768) (Figure 4B). When comparing CCS measurements in males, the difference was even less pronounced (*p* = 0.4897) (Figure 4B).

**Figure 5:**
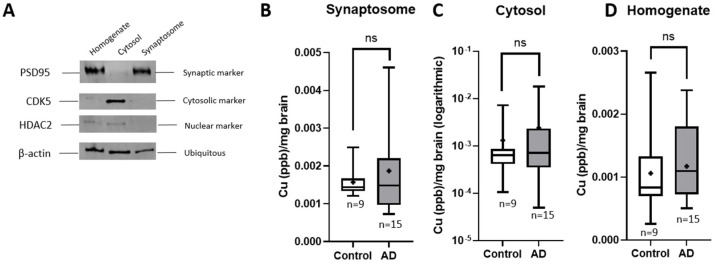
(**A**) Human brain tissue total homogenate, isolated homogenate fraction, cytosol fraction, and synaptosomal suspension confirmation via Western blot. The full membrane is available to view in Supplemental Figure S5. (**B**–**D**) ICP-MS runs on each isolated fraction show no significant changes in total copper levels in any fraction (synaptosomes *p* = 0.3869, cytosolic *p* = 0.4461, homogenate *p* = 0.6939). For each graph, n = 9 for controls and n=15 for AD cases. Lines represent the median value, and the means are represented by +. A representation of these data as scatter dot plots is shown in Supplemental Figure S6. AD, Alzheimer’s disease; ICP-MS, inductively coupled plasma mass spectrometry.

To investigate copper redistribution in AD brains, we separated the brain tissues into three neuronal compartments: synaptic, cytosolic, and general homogenate. This fractionation approach allowed us to directly measure the total copper levels in distinct cellular regions and evaluate potential dysfunction in the copper chaperones responsible for maintaining proper copper homeostasis. As shown in Figure 5A, the cytosolic marker CDK5 was detected exclusively in the cytosolic fraction, as expected. Similarly, the nuclear marker HDAC2 was absent from the synaptosomal fraction, confirming proper fractionation. The synaptic marker PSD95 was prominently represented in the synaptosomal fraction and, to a lesser extent, in the general homogenate, further validating the accuracy of the separation process. Raw data for this figure are shown in Supplemental Figure S5. After confirming successful fractionation, the individual fractions were analyzed for total copper content using ICP-MS (Figure 5B–D), as described in the Materials and Methods section. Analyses of each neuronal fraction using a two-tailed *t*-test with Welch’s correction revealed no statistically significant differences in copper levels between the two populations across the synaptic, cytosolic, or homogenate fractions. (Synaptosomes *p* = 0.3869, cytosolic *p* = 0.4461, homogenate *p* = 0.6939.)

**Figure 6:**
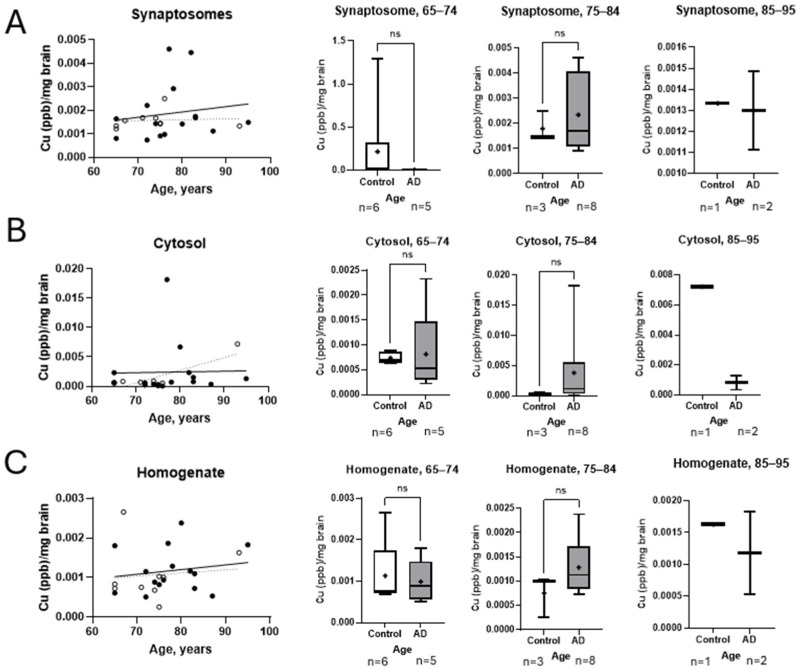
Age-stratified analysis of copper levels in neuronal subcellular fractions. (**A**) No significant differences were seen in copper levels in the synaptosomal fraction across age groups. (**B**) Control cohorts exhibited a trend toward elevated cytosolic copper with advancing age, while AD patients showed a plateau or decline in copper levels. Differences between groups did not reach statistical significance. (**C**) No significant differences (ns) were seen in the general homogenate. No differences were observed across all age groups. Lines represent the median values, and the means are represented by the symbol +. AD, Alzheimer’s disease; Cu, copper.

To further refine our findings, we analyzed copper measurements across different age groups to identify any subtle trends. As shown in Figure 6, both synaptosomal and homogenate copper levels followed similar patterns with minimal overall change across age groups. The copper levels in both fractions show no significant difference, suggesting that age alone does not significantly influence copper redistribution in this dataset (Figure 6A,C). Copper in the cytosolic fraction sees an upward trend with advancing age in control cases, whereas the AD population stays relatively stable (Figure 6B).

**Figure 7:**
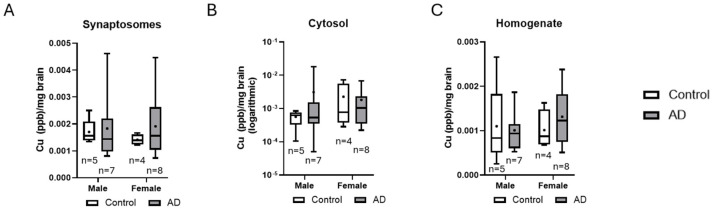
Copper measurements in isolated neuronal fractions were compared between male and female patients and between diagnoses, with lines representing the median values and the means represented by the symbol +. Sex did not have a statistically significant effect on copper measurements in synaptosomes (**A**), cytosolic (**B**), and homogenate layers (**C**). Scatter dot plots of these data are available in Supplemental Figure S7. AD, Alzheimer’s disease; Cu, copper.

When comparing copper levels by sex, no significant differences were found between men and women across any fraction (Figure 7). This observation suggests that sex does not significantly affect copper distribution within neurons when comparing AD cases to non-diagnosed individuals.

## 4. Discussion

Our study uncovered a correlation between CCS levels and advanced AD diagnosis. This finding sheds light on a critical aspect of cellular copper management in AD. Normally, cells maintain very low levels of free intracellular copper to prevent oxidative damage. When this balance is disrupted and copper accumulates, it can rapidly cycle between its Cu(II) and Cu(I) states in a Fenton-type reaction to generate ROS at much higher rates, potentially overwhelming the antioxidant defenses of the cell [11,43]. Under normal conditions, copper presence promotes SOD1 Cu binding [44,45,46], and the failure of SOD1 to incorporate copper has been observed in CCS-/- mice [47]. Therefore, the loss of functional CCS triggers chronic inflammation and exacerbates ROS accumulation as declining oxidative defenses (including glutathione and GPX4) are overwhelmed by the radical products of free copper ions [22,48,49]. This highlights a potential mechanism by which copper chaperone dysregulation contributes to AD pathology and warrants further investigation.

Interestingly, our analysis of secondary factors revealed a trending divergence in CCS levels with advancing age. While CCS levels were similar between the AD and control groups at around 65 years old, a measurable decline in CCS was observed in older AD patients as they approached their 90s (Figure 3B and Figure 4). This contrasts with the slight increase in CCS levels seen in the control group, suggesting that rather than a systemic decrease across the entire population (as Figure 2C might imply), CCS reduction becomes more pronounced with age specifically in AD patients, possibly contributing to the oxidative stress and cellular damage characteristics of AD [50]. In healthy cases, CCS expression may increase to boost antioxidant defenses, counteracting the oxidative stress caused by copper accumulation seen with normal aging to maintain neuronal health.

In our analysis of the effects of sex on copper chaperones, we observed an intriguing trend in CCS levels among AD patients, with female AD patients exhibiting lower CCS levels compared to controls, with a more pronounced difference than that seen in male patients. Although this difference did not reach statistical significance with our current sample size, the disparity in *p*-values between females (*p* = 0.0768) and males (*p* = 0.4897) suggests that a larger study cohort might reveal a significant sex-dependent effect (Figure 4B). There is no consensus in the literature confirming CCS differences between sexes. Limited human data do show that zinc supplementation (150 mg per day) reduces serum ceruloplasmin and erythrocyte SOD activity in females, suggesting a sex-dependent susceptibility to dietary copper deficiency [51]. Interestingly, studies in mouse models suggest that CCS deficiency can be reversed with copper supplementation [52,53]. While the literature on the effects of sex on neuronal CCS levels is limited, some evidence indicates that female mice experience more severe copper deficiencies than males [54]. The trending decline we observed in CCS levels in female AD patients implies that this effect may be stronger in women for reasons not yet understood. Seeing as AD is more prominent in women, this finding may point towards a correlation in this disparity [55]. Further work needs to be carried put to confirm if this is more than a trend, highlighting the need for further research to explore sex-specific mechanisms underlying copper metabolism and CCS regulation in AD.

Our analysis of copper distribution in AD brains revealed intriguing age-related patterns, despite finding no significant differences in cytosolic copper levels between AD patients and non-diagnosed controls (Figure 5C). Notably, we observed a trending increase in cytosolic copper levels with age in control subjects, a pattern absent in AD individuals (Figure 6B). In normal aging, this increasing trend in cytosolic copper might be counterbalanced by a corresponding increase in CCS levels, potentially mitigating ROS production associated with elevated free copper. The absence of this trend in AD patients suggests a possible disruption in this compensatory mechanism.

Our findings on DCTN4 and synaptosomal copper levels in AD brains were unexpected. Unlike previous studies, we did not observe significant changes in DCTN4 levels in AD brains compared to controls. This result is surprising, as earlier research reported decreased DCTN4 in AD brains, suggesting impaired copper transport to synapses. Our contrasting findings raise new questions about the role of DCTN4 in AD and highlight the complexity of copper homeostasis in neurodegenerative diseases [56].

Our analysis of DCTN4 levels in relation to age revealed similar upward trends in both control and AD cases among older patients. This stability in DCTN4 levels corresponded with a lack of copper decline in the synaptosomal fraction, suggesting that copper transport to synapses may not be as impaired in AD as previously thought. These findings raise questions about the role of DCTN4 in copper homeostasis and its contribution to AD pathology. One interpretation is that DCTN4 continues to facilitate copper transport in AD brains but may not upregulate it sufficiently to meet the increased demands caused by elevated free copper and neuronal stress. This limited adaptability could contribute to oxidative overload, potentially exacerbating AD progression. Additionally, there was a decreasing trend in female AD patients compared with almost no difference in men. The literature is not clear on any DCTN4 differences regarding sex, especially with regard to AD, and the relevance of these findings remains to be explored in the future.

The literature suggests that ATOX1 is under expressed in AD cases [50,57], although our work here revealed no significant changes in ATOX1 protein, including expression with regard to age and sex. We speculate that as CCS declines, any significant changes leading to copper overload may activate alternative pathways to help maintain homeostasis. ATOX1 may be successfully directing the copper pool towards pathways for excretion. This may also help explain why we are not seeing a significant surge in copper in the cytosolic fraction, as we would have expected. Another possibility is that ATOX1 is decreased in regions outside of Brodmann’s area 21 or is more visible when looking at different specific cell types, such as astrocytes and oligodendrocytes. One further possibility is that ATOX1 leads to a significant decrease in AD cases with cerebral amyloid angiopathy present as a comorbidity, while this study specifically looks at AD-only diagnoses. Further studies would be required to illuminate any potential differences.

This study has several limitations that should be acknowledged. It focuses on elderly AD patients, a subset of the broader AD population. Since many AD cases also involve varying severities of cerebral amyloid angiopathy [58], future studies should include these comorbidities to provide a more comprehensive understanding of changes across the general AD population. Additionally, this study used a Caucasian-only cohort to minimize potential genetic variables, but this approach also limits insights into demographic and genetic diversity. Further research is needed to explore differences across AD subtypes, including early-onset cases and younger patients. Another limitation of our study is the absence of compartment-specific protein quantitation within neuronal subfractions. For instance, while ATOX1 is ubiquitously expressed across cellular compartments—including the cytosol, nucleus [59], and peri-Golgi regions [60]—its functional redistribution between these domains could critically influence copper trafficking, even if total ATOX1 levels remain unchanged in AD. Such spatially resolved dynamics may underlie pathogenic copper mislocalization, such as mitochondrial accumulation or synaptic depletion, which our bulk analyses could not resolve. Finally, the limited availability of brain tissue may have resulted in underpowered analyses. Some findings were close to statistical significance, suggesting that increasing the sample size could yield more robust and well-defined results. These limitations highlight the need for further studies to refine and expand upon this work.

## 5. Conclusions

Our investigation into copper chaperone dynamics in AD revealed a pronounced correlation between reduced levels of the copper chaperone for CCS and AD pathology, underscoring CCS’s potential role in neuronal copper transport. Notably, we observed a suggestive sex-based trend, with female AD patients exhibiting a more pronounced decline in CCS levels compared to males, though this finding did not reach statistical significance. Furthermore, neuronal fractionation analyses indicated a tendency toward elevated cytosolic copper in older AD patients, aligning with aging-related copper dyshomeostasis hypotheses, but again without statistical significance.

While the majority of copper localization changes lacked statistical robustness, the consistent trends across cohorts highlight a compelling need for expanded studies to unravel the mechanistic links between CCS depletion, ROS generation, and AD pathogenesis. Future research should prioritize larger, sex-balanced cohorts and advanced spatial profiling techniques to clarify whether CCS loss exacerbates copper-mediated oxidative stress or serves as a compensatory response in AD progression. These insights could pave the way for novel therapeutic strategies targeting copper homeostasis in neurodegeneration.

## Figures and Tables

**Table 1:** Demographics of brain samples used. An average of 0.975 g of each sample was obtained. All samples were from Brodmann’s Area 21. All AD patients had a Braak and Braak score of VI. Additional information on all samples available in Supplemental Table S1.

Case	Age Range	n		Diagnosis	BB
Diagnostic pathology not present	65–93	Male Female Total	9 4 13	Control	-
Alzheimer’s Disease	65–95	Male Female Total	7 8 15	AD	VI

## Data Availability

No new datasets were generated. Raw data are presented in Supplementary Materials.

## Supplementary Materials

The following supporting information can be downloaded at: https://www.mdpi.com/article/10.3390/brainsci15030216/s1, Table S1: Brain samples information; Figure S1: Complete Western membranes used in Figure 2; Figure S2: Scatter dot plots for copper chaperones between control and AD cases; Figure S3: Copper chaperone data grouped by age range; Figure S4: Scatter dot plots for copper chaperones grouped by sex; Figure S5: Unedited membrane for neuronal fractionation; Figure S6: Scatter dot plots for copper measurements in neuronal fractions; Figure S7: Scatter dot plots for copper in neuronal fractions grouped by sex.

## Abbreviations

The following abbreviations are used in this manuscript:

AD	Alzheimer’s disease
ANOVA	Analysis of variance
ATP7A	ATPase copper transporting alpha
ATP7B	ATPase copper transporting beta
ATOX1	Antioxidant protein 1
CCS	Copper chaperone for superoxide dismutase
CDK5	Cyclin-dependent kinase 5
COX17	Cytochrome c oxidase copper chaperone 17
CTR1	Copper transporter 1
Cu	Copper
Cul3	Cullin 3
DCTN4	Dynactin subunit 4
DMT1	Divalent metal transporter 1
EDTA	Ethylenediaminetetraacetic acid
EGTA	Ethylene glycol-bis(β-aminoethyl ether)-N,N,N′,N′-tetra acetic acid
ELISA	Enzyme-linked immunosorbent assay
GPx	Glutathione peroxidase
HDAC2	Histone deacetylase 2
ICP-MS	Inductively coupled plasma mass spectrometry
Keap1	Kelch-like ECH-associated protein 1
NMDA	N-methyl-D-aspartate
NIST	National Institute of Standards and Technology
Nrf2	Nuclear factor erythroid 2-related factor 2
PSD95	Postsynaptic density protein 95
ROS	Reactive oxygen species
SDS	Sodium dodecyl sulfate
SOD1	Superoxide dismutase 1
SOD3	Superoxide dismutase 3
STEAP	Six transmembrane epithelial antigen of prostate
Tris	Tris(hydroxymethyl)aminomethane
