# Supplementary material for: Downregulation of Parahippocampal Copper Chaperone for Superoxide Dismutase in Alzheimer’s Disease
Source: Brain Sci. 2025 Feb 20;15(3):216. doi: 10.3390/brainsci15030216 (PMC11940324; doi:10.3390/brainsci15030216)
Supplement: Supplementary file 1 [file brainsci-15-00216-s001.zip › brainsci-3485394-supplementary.pdf]

brain sciences

MDPI

Table S1. Brain samples used. All come from Brodman's Area 21. Samples diagnosed with Alzheimer's disease are categorized at Braak & Braak Stage VI. Tissue specimens were obtained from the BIH Brain & Tissue Repository-California, Human brain & Spinal Resource Cetner, VA.

HSB#	AGE	SEX	NEUROPATHOLOGY DIAGNOSIS	CORONAL SLAB #	AUTOLYSI S T	GRAM S
**5468**	65	M	Diagnostic pathology not present	9	28.8	1.1
**5568**	65	F	Diagnostic pathology not present	9	22.4	0.8
**3236**	67	M	Diagnostic pathology not present	10	16.3	1.2
**3606**	71	M	Diagnostic pathology not present	11	11.5	0.5
**5718**	72	M	Diagnostic pathology not present	9	11.5	1
**3603**	74	F	Diagnostic pathology not present	11	12	0.9
**1718**	75	F	Diagnostic pathology not present	9	9.8	0.9
**3590**	75	M	Diagnostic pathology not present	8	11.5	0.8
**3586**	76	M	Diagnostic pathology not present	9	16	1
**3750**	77	M	Diagnostic pathology not present	9	12.3	1
**3777**	84	M	Diagnostic pathology not present	9	13.5	0.7
**5609**	87	M	Diagnostic pathology not present	9	23.2	1.5
**3465**	93	F	Diagnostic pathology not present	11	20.3	1.1
**1802**	65	F	Alzheimer's disease	7	6.5	0.8
**2642**	65	M	Alzheimer's disease	9	7.5	1
**1829**	72	F	Alzheimer's disease	8	4	1
**2748**	74	M	Alzheimer's disease	8	8.5	0.9
**1823**	75	M	Alzheimer's disease	10	6.8	1
**3367**	75	F	Alzheimer's disease	11	19.8	1
**3512**	76	M	Alzheimer's disease	9	13.8	1.1
**3121**	77	M	Alzheimer's disease	9	16	0.7
**3377**	78	F	Alzheimer's disease	11	11	1.1
**1607**	80	F	Alzheimer's disease	9	5.5	1.2
**1660**	82	F	Alzheimer's disease	9	18	1.2
**3027**	83	F	Alzheimer's disease	10	7	1.3
**4108**	83	M	Alzheimer's disease, probable	9	16.5	0.6
**1773**	87	M	Alzheimer's disease	10	9	1.1
**2313**	95	F	Alzheimer's disease	10	7.5	0.8

 https://doi.org/10.3390/brainsci15030216

West Los Angeles Medical Center, Los Angeles, California, which is supported in party by National Institutes of Health and the US Department of Veterans Affairs. This brain bank operates under the IRB Protocol Number PCC#: 2015-060672, VA Project #: 0002, managed by the Department of Veterans Affairs – Los Angeles.

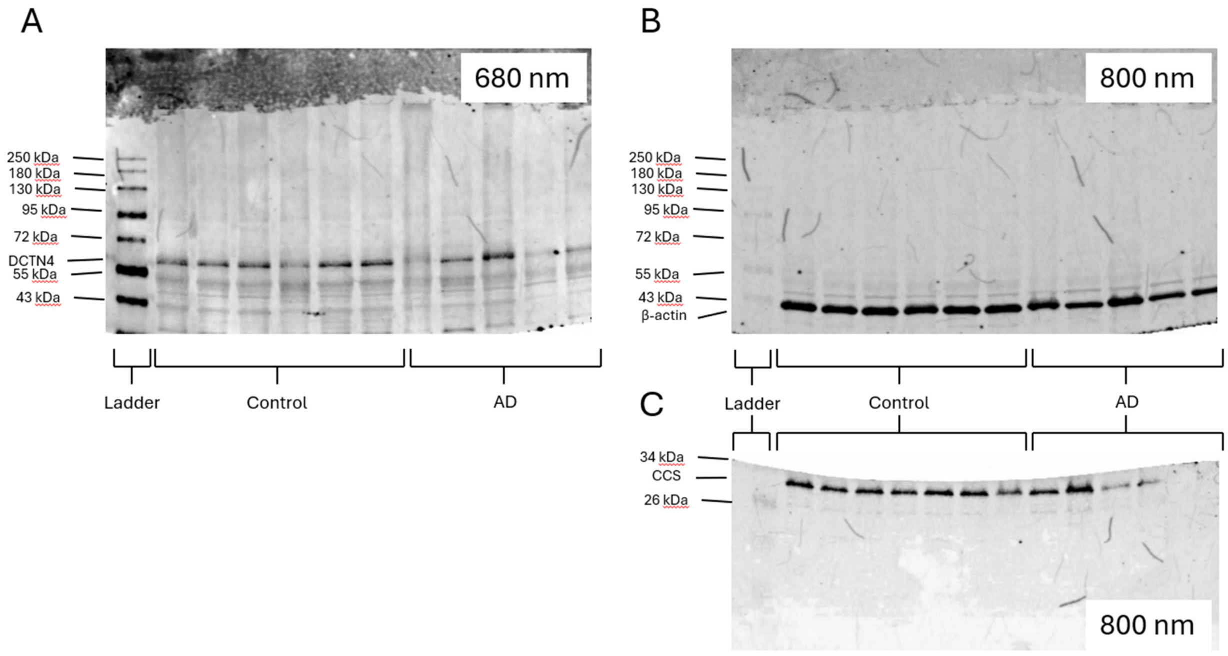

Figure S1. Complete western blot shown in sections in Figure 2. The membrane was cut partway through to ensure clarity in antibody binding by physical separation during primary incubation. Bands were measured against Cell Signaling Technology Color-coded Prestained Protein Marker, Broad Range (10-250 kDa) #74124. **A**. Upper half staining for DCTN4 seen at 680nm. **B**. Upper half staining for β-actin using 800nm channel. **C.** Lower half staining for CCS using 800nm channel.

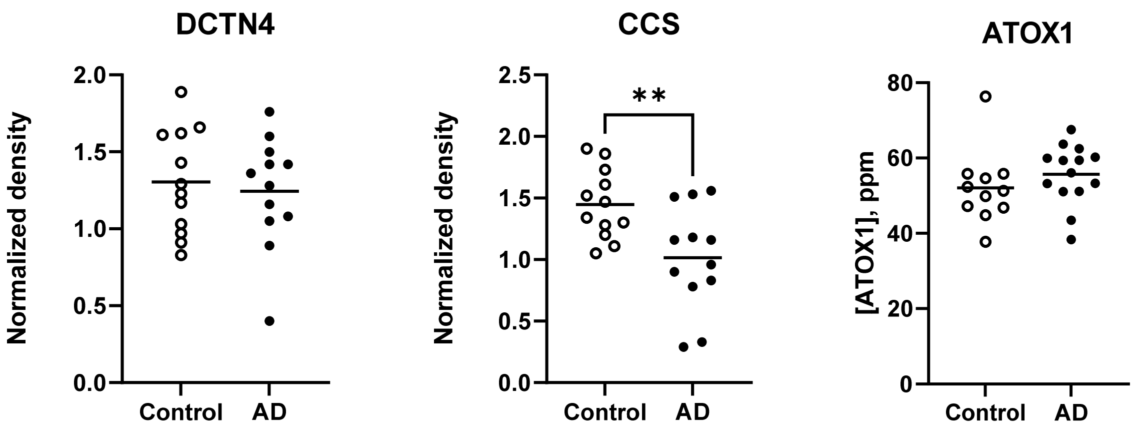

Figure S2. Data shown in Figure 2B-C as box plots are displayed here as scatter dot plots to show individual data points. Horizontal lines represent mean values. DCTN4 and CCS were averaged from western blot experiments, calculated using ImageJ and Microsoft Excel as outlined by Stael et al. ATOX1 was measured using competitive ELISA kit (MyBioSource.com, MBS7217810) and calculated using a 4-parameter logistic regression. Statistical analysis was done using Welch's t-tests via GraphPad Prism 10.3.1. Plots were generated using this software. No significant changes in chaperone expression were seen for DCTN4 ($p = 0.6797$) or ATOX1 ($p = 0.3318$). CCS was significantly reduced in AD brains ($p = 0.0085$).

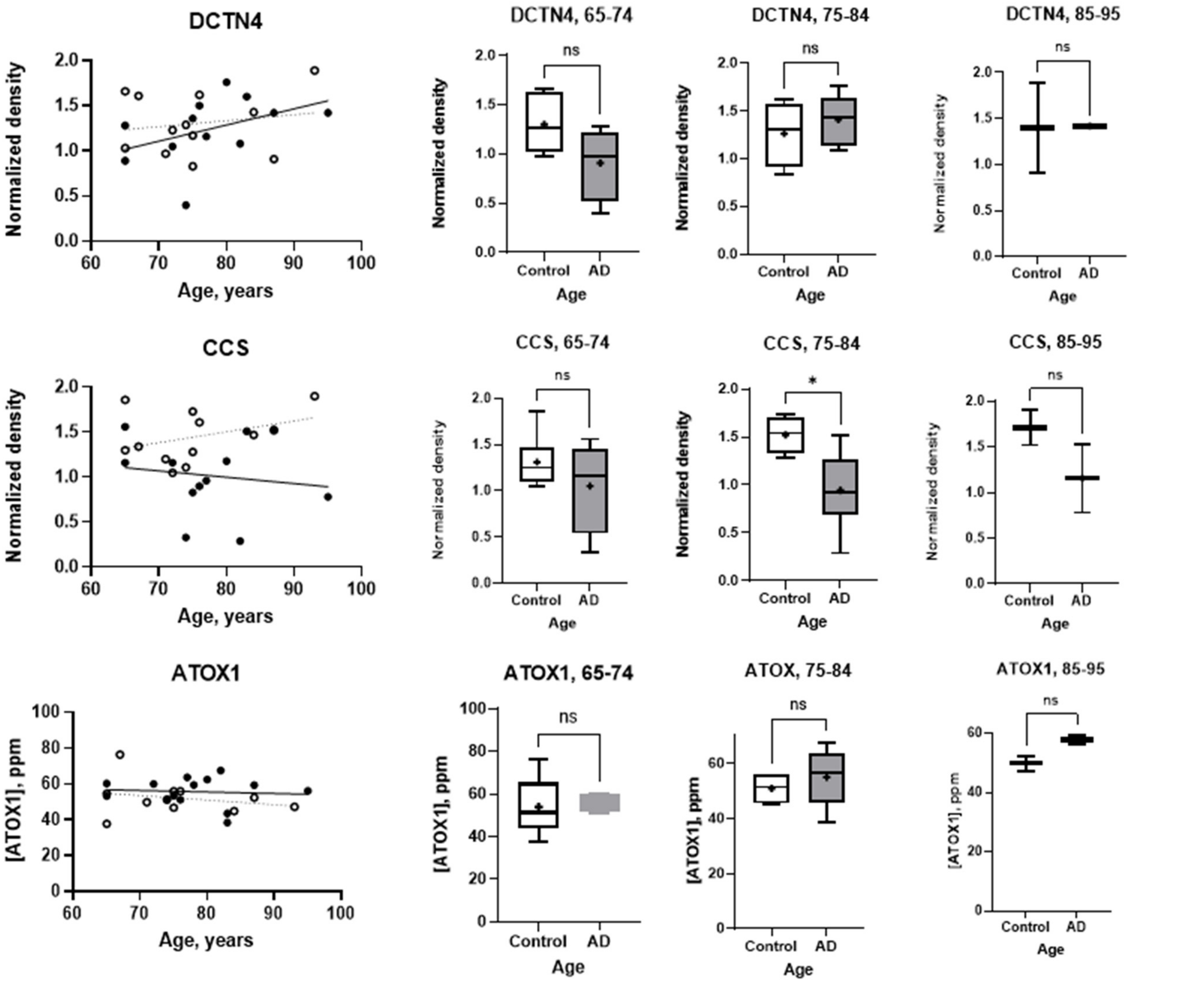

Figure S3. Data shown in Figure 3, each divided into three age groups: 65-74, 75-84, and 85-95. Each chart for DCTN4 and ATOX1 shows no significant difference between the control and AD samples when compared across each age bracket. Splitting CCS into the three age brackets illustrates a trend of increasing significant changes in measurements of the copper chaperone, with none seen in 65-74 years, significance shown for 74-85 with p = 0.0178, and none seen in 85-95 (p = 0.3544) due to the lower number of samples in that age bracket (n = 2 for both control and AD cases).

.

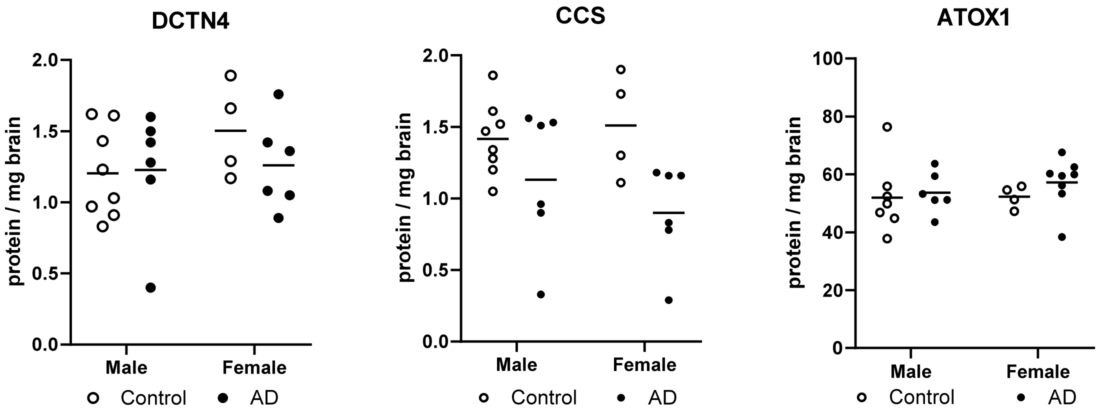

Figure S4. Data shown in Figure 4 as scatter dot plots to show individual data points. Horizontal lines represent mean values. DCTN4 and CCS were averaged from western blot experiments, calculated using ImageJ and Microsoft Excel as outlined by Stael et al. ATOX1 was measured using competitive ELISA kit (MyBioSource.com, MBS7217810) and calculated using a 4-parameter logistic regression. Statistical analysis done using Microsoft Excel Statistical analysis was done using 2-way ANOVA via GraphPad Prism 10.3.1. Plots were generated using this software. No significant changes were observed making multiple comparisons with sex and diagnosis. The closest was female control cases compared against female AD cases, with P = 0.0768. Sex was not a significant variable.

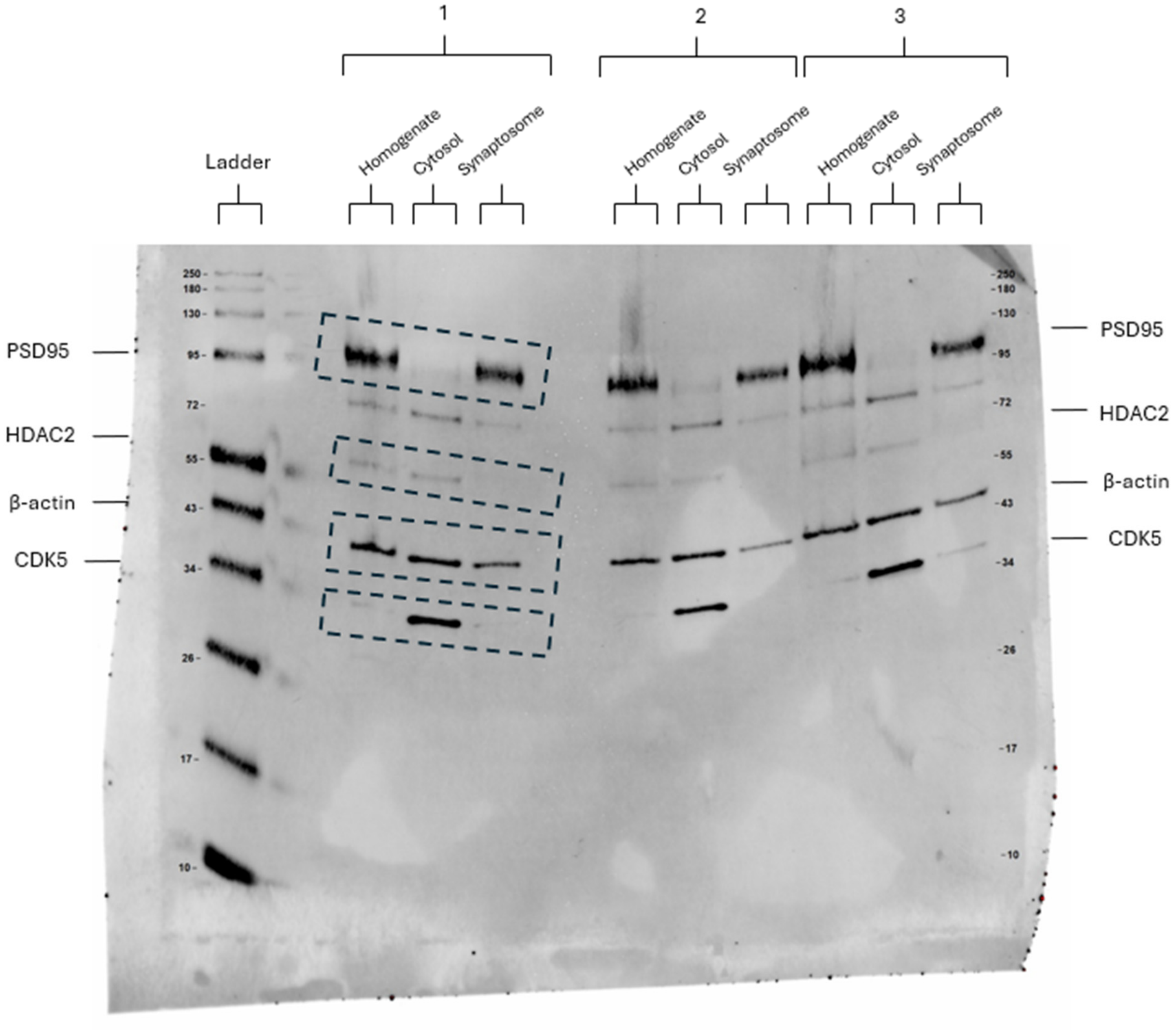

Figure S5. Unedited membrane for Figure 5A. Synaptosomal fraction western blot. Synaptosomal fraction was made using Thermo Fisher's Syn-PER isolation buffer. Primary antibodies were used for synaptic marker PSD95 (95 kDA), cytosolic marker CDK5 (33 kDa), nuclear marker HDAC2 (55 kDa), and B-actin (42 kDa) was used for loading marker. Synaptic markers were observed reliably in the synaptosomal suspension, with PSD95 absent in cytosolic layer as expected. CDK5 shows up in all layers as expected. Experiment was done in triplicate. The boxes show which bands were used in Figure 5A.

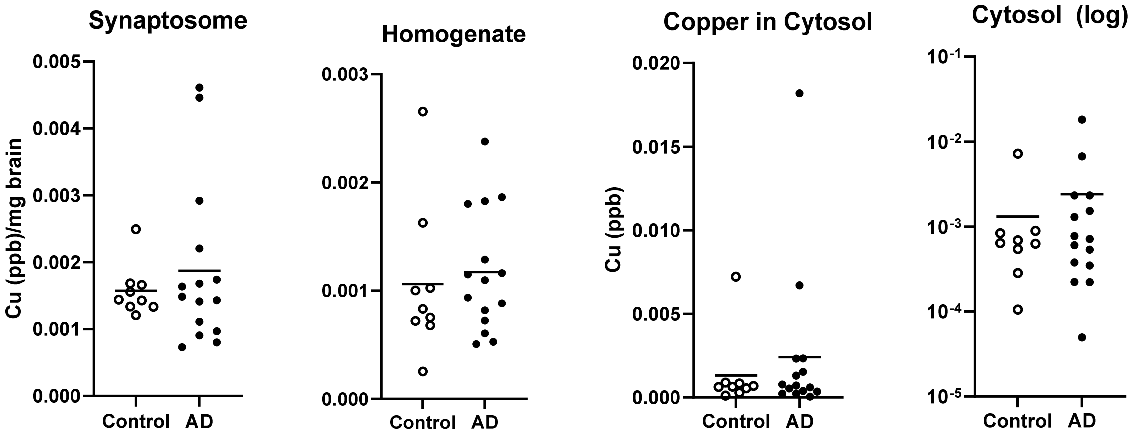

Figure S6. Data shown in Figure 5B-D as box pots are shown here as scatter dot plots to show individual data points. Horizontal lines represent mean values. Fractions were analyzed by ICP-MS equipment at UC Riverside, with analysis done using Microsoft Excel Statistical analysis was done using Welch's t-tests via GraphPad Prism 10.3.1. Plots were generated using this software. No significant changes in copper presence in the synaptosome fraction (P = 0.3869), the homogenate (P = 0.6939), or the cytosolic fraction (P = 0.4461). The cytosolic fraction is displayed twice here, once with a linear Y-axis and again with a logarithmic Y-axis for visual clarity. The box plot based on the logarithmic chart was used in Figure 5C.

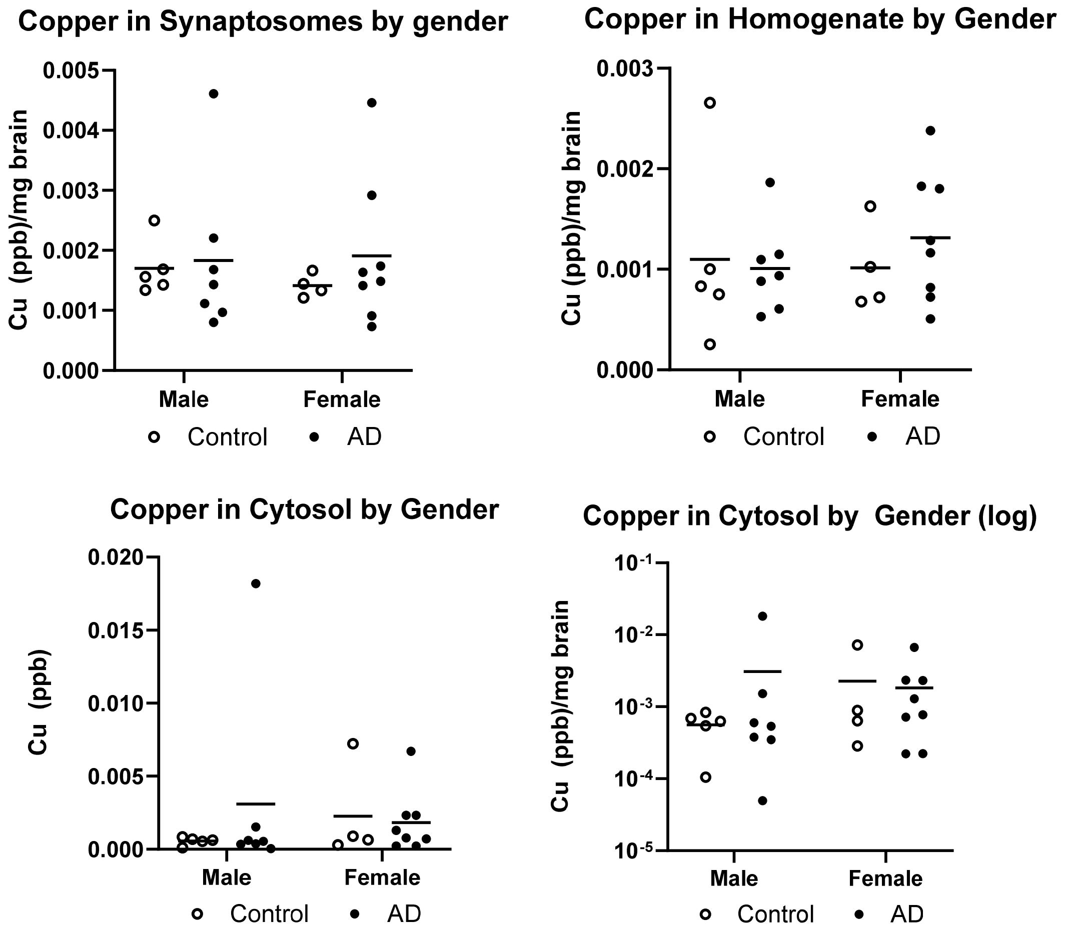

Figure S7. Data shown in Figure 7 as scatter dot plots to show individual data points. Horizontal lines represent mean values. Fractions were analyzed by ICP-MS equipment at UC Riverside, with data processing done using Microsoft Excel. Statistical analysis was done using 2-way ANOVA via GraphPad Prism 10.3.1. Plots were generated using this software. There was no significant difference seen when looking at sex in any analysis of copper distribution. The cytosolic fraction is displayed twice here, once with a linear Y-axis and again with a logarithmic Y-axis for visual clarity. The box plot based on the logarithmic chart was used in Figure 6E. We conclude that sex was not a significant variable.
